# The optimal time for laparoscopic excision of ovarian endometrioma: a prospective randomized controlled trial

**DOI:** 10.1186/s12958-023-01109-2

**Published:** 2023-06-27

**Authors:** Qing Wu, Qingmei Yang, Yanling Lin, Lin Wu, Tan Lin

**Affiliations:** 1 Reproductive Medicine Center, Department of Gynecology, Affiliated People’s Hospital, Zhejiang Provincial People’s Hospital, Hangzhou Medical College, Hangzhou, Zhejiang, 310014 P.R. China; 2 Department of Obstetrics and Gynecology, Fujian Provincial Hospital, Clinical Medical School of Fujian Medical University, Fuzhou, 350001 Fujian P.R. China; 3 Department of Clinical Laboratory, School of Medicine, Xiang’an Hospital of Xiamen University, Xiamen University, Xiamen, 361101 China

**Keywords:** Laparoscopy, Endometriosis, Ovarian reserve, AMH, Menstrual cycle

## Abstract

**Objective:**

This study aimed to explore the optimal time of laparoscopic cystectomy for unilateral ovarian endometrioma patients and evaluate the influence on ovarian reserve.

**Materials and methods:**

This prospective randomized controlled study included 88 women with unilateral ovarian endometrioma at a tertiary teaching hospital. All patients received their first identified diagnosis of ovarian endometrioma by ultrasound (> 4 cm and ≤ 10 cm) and were administered an oral contraceptive pill (OC) for one cycle before laparoscopy. They were randomly divided into two groups: laparoscopy at the late luteal phase (group LLP) (n = 44) (termination of OC for two days) and laparoscopy at the early follicular phase (group EFP) (n = 44) (day 3 after menstruation). Basic clinical characteristics were recorded. Serum Anti-Müllerian hormone (AMH) levels were measured at various times to predict ovarian reserve. Serum levels of Anti-Müllerian hormone (AMH) were measured at several time sites to predict the ovarian reserve; AMH and leukocyte esterase (LE) levels of the endometrioma wall were measured.

**Results:**

Before surgery, serum AMH levels decreased in both groups from preoperative to one week and six months postoperatively. In contrast, the difference values of group EFP were larger than those of group LLP at postoperative one week and postoperative six months (1.87 ± 0.97 vs. 1.31 ± 0.93, *P* = 0.07; 1.91 ± 1.06 vs. 1.54 ± 0.93, *P =* 0.001). The mean rates of postoperative serum AMH decline were 37.92% and 46.34% in group EFP, significantly higher than those in group LLP (25.83% vs. 31.43%, *P* < 0.001). Ovarian endometrioma wall AMH of group LLP was significantly lower than that of group EFP ([22.86 ± 3.74] vs. [31.02 ± 5.23], P < 0.001). Meanwhile, ovarian endometrioma LE concentration of group LLP was significantly higher than that of group EFP ([482.83 ± 115.88] vs. [371.68 ± 84.49], *P*<0.001). There was also a significant inverse correlation between leukocyte esterase and AMH concentration in an ovarian endometrioma cyst wall (r=-0.564, *P*<0.001).

**Conclusion(s):**

The optimal time for laparoscopic cystectomy for patients with first identified unilateral ovarian endometrioma is the late luteal phase, which reduces ovarian tissue loss and preserves ovarian reserve effectively and safely.

## Introduction

Endometriosis (EMT) is a common gynecological condition characterized by endometrial tissue outside the uterine cavity resulting in dysmenorrhea, chronic pelvic pain, pelvic masses, and infertility, which can seriously affect a woman’s health and quality of life. Ovarian endometriomas are the most common type of EMT, with a prevalence of 17–44% in patients with endometriosis [1]. Laparoscopic cystectomy has become the gold standard in the surgical management of persistent adnexal masses, including ovarian endometriosis, with the surgical aim of removing all visible endometriosis lesions and restoring anatomy [2]. However, ovarian cystectomy may harm ovarian reserves [3–5]. In addition, surgical procedures on the ovaries lead to ovarian tissue damage, which can strip normal ovarian tissue and exacerbate the harm to the remaining follicles, raising concerns of gynecologists regarding the use of different surgical procedures in this field [6, 7]. However, only a few studies have focused on the optimal time of surgery for ovarian endometrioma.

Anti-Müllerian hormone (AMH) is produced by the granulosa cells of primary, preantral, and small antral follicles, not primordial ones. Therefore, AMH level indirectly represents the quantity of the ovarian follicle pool, estimated by the number of early growing-stage follicles. Moreover, serum AMH levels appear independent of the menstrual cycle and are unaffected by gonadotropin-releasing hormone (GnRH) agonists or oral contraceptives [8–11]. Therefore, serum AMH levels, as a promising and reliable parameter, have been used to assess the ovarian reserve around treatments that potentially cause ovarian damage [12–15].

Currently, no precise data exist on whether laparoscopic endometrial cystectomy with different menstruation phases reduces the damage to ovarian function, shortens the operation time, reduces intraoperative blood loss, and accelerates patient recovery. Therefore, this study aimed to explore the optimal timing of the first laparoscopic cystectomy in ovarian endometrioma patients with unilateral and evaluate the influence on the patient’s ovarian reserve.

## Materials and methods

This prospective clinical study was approved by the board of Fujian provincial hospital ethics committee (2018ky0024) and registered under the clinical trial registry number (ChiCTR1800019766). All patients provided preoperative informed consent after being informed of potential risks and complications. All patients provided preoperative informed consent after being informed of potential risks and complications. In total, 88 patients with unilateral ovarian endometrioma were recruited into this prospective study at the Department of Obstetrics and Gynecology in Fujian provincial hospital from March 2019 to March 2021. After inclusion in the study, all patients were administered an oral contraceptive pill (OC, drospirenone, and ethinylestradiol) for one cycle to determine the timing of surgery. Inclusion criteria were as follows: (1) age 20–36 years; (2) regular menses; (3) clinical and ultrasonographic finding of unilateral ovarian endometrioma ≥ 4 cm and ≤ 10 cm the first time; (4) without pregnancy or plan to get pregnant in six months. Exclusion criteria were as follows: (1) any suspicious finding of malignant ovarian diseases; (2) ovarian, uterine, or tubal surgery history; (3) endocrine disease and treatment history; (4) long-term use of hormonal drugs for more than three months (e.g., gonadotropin-releasing hormone analogs); (5) smokers. Patients with infiltrated endometriosis were excluded from this study based on transvaginal ultrasound and gynecological examination. Patients who fulfilled the inclusion criteria and consented to participate in the study were enrolled. The study objectives and steps were explained to all patients before enrollment. All experimental procedures were performed following the guidelines for the Declaration of Helsinki. Our study was conducted according to the CONSORT guidelines [16].

### Sample size calculation

Using a two-sided equal-variance t-test, group sample sizes of 30 and 30 achieved 81.328% power to reject the null hypothesis of equal means when the mean population difference was 1.13. The standard deviation for both groups was 1.51, and the significance level (alpha) was 0.05 [3, 17]. Furthermore, considering the probability of dropouts during follow-up, the number of cases was further increased to more than 40 patients. The sample size was estimated using G*Power© software (Institutfür Experimentelle Psychologie, Heinrich Heine Universität, Düsseldorf, Germany) version 3.1.9.2.

### Randomization

All patients were diagnosed with ovarian endometrioma by ultrasound and were administered OC for one cycle before laparoscopy to inhibit ovulation and identify the menstruation phase. After written consent, the randomized number was concealed in an opaque, sealed envelope for each patient, and the envelopes were opened sequentially by a study nurse before surgery. Randomization was performed in a 1:1 ratio, according to a computer-generated number list, into two groups. The single number drawn was included in the group LLP, and the double number was included in the group EFP. All patients were randomly divided into two groups: laparoscopy at late luteal phase (group LLP) (n = 44): termination of OC for two days; and laparoscopy at Early follicular phase (group EFP) (n = 44): day 3 after menstruation.

### Surgical technique

All surgeries were performed by the same surgeons with extensive experience in endometriosis surgery, who were particularly aware of the necessity to avoid damaging or removing healthy ovarian tissue. Surgeons were blinded to the result of group Randomization. Laparoscopic pneumoperitoneum was induced by CO_2_ insufflation using a laparoscopic Veress needle. Umbilical 10-mm trocar and laparoscope entries were performed. Another three trocars were inserted through lower abdominal incisions under direct laparoscopic vision. If peri-ovarian adhesion and adhesion of Douglas fossa were present, Blunt dissection and sharp separation were combined to detach the adhesion. After mobilization of the cystic adnexa, ovarian cystectomy was performed by incising the cyst with cold scissors and carefully identifying, separating, and completely removing the entire cystic wall from the ovarian cortex by traction/counter traction using non-traumatic grasping forceps. Hemostasis was achieved using 3 − 0 absorbable sutures that were carefully selected (Vicryl; Ethicon Inc., New Jersey, USA) without electrocoagulation devices. Blood loss was estimated by combining the volume of blood collected within the suction canister with the gauze weight used during surgery. The endometriosis stage was determined based on the revised classification of the American Society of Reproductive Medicine (r-ASRM) [18].

### Hormonal assays

All patients provided serum specimens prior to anesthesia, as well as at one week and six months following the procedure. Venous blood samples were obtained, and serum was extracted by centrifugation. According to manufacturer’s instructions, serum E2 and P levels were measured by enzyme-linked fluorescent assay (ELISA; Beckman Coulter Inc., Ireland). Serum AMH level was measured by a commercially available enzyme-linked immunosorbent assay kit (ELISA; Beckman Coulter Inc., Ireland) and reported as nanograms per milliliter with a detection limit of 0.16 ng/mL. Postoperative serum AMH level and AMH decline were the primary outcome measures.

### Tissue sample collection

After a naked-eye examination of the entire cyst wall, five pieces of the specimen of 5 mm^2^ were obtained from cyst walls at different portions. One was from the intermediate part of the specimen, and the others were from the four quadrants. Other cyst walls were sent to the pathology laboratory, and a pathologic examination confirmed the ovarian endometriosis diagnosis. Leukocyte esterase concentration and tissue AMH levels in the cyst wall were measured using (LE/AMH) ELISA kit (Jiangsu Meimain Co., Ltd., Jiangsu, China) as secondary endpoints. All hormonal measurements were performed at the same laboratory.

### Unilateral ovarian involvement

We compared the potential role of unilateral ovarian involvement on preoperative levels and postoperative changes in AMH values after laparoscopic endometrioma excision. AMH decline (% decline AMH) was used to compare the changes in AMH levels in endometrioma resected at different menstrual cycles. The rate of AMH decline was calculated using the following formula: (% decline AMH) = (preoperative AMH level – AMH at one week or six months postoperatively)/preoperative AMH level.

### Statistical analysis

Categorical variables are described using proportions. Baseline patient characteristics were calculated via t-test for comparisons of normally distributed data and the rank-sum test for comparisons of non-normally distributed data. Count data were summarized as percentages and compared using chi-square and Fisher’s exact tests. Analysis of variance (ANOVA) was used in intra-group comparison at different time points. A two-sided *P*-value of less than 0.05 was considered to be significant. The relationship between ovarian endometrioma wall AMH and leukocyte esterase concentration were generated based on significant Pearson correlations between data. Statistical significance was set at p-value < 0.05. All data were analyzed using SPSS version 26 (IBM Corp., Armonk, NY, USA) and PRISM version 9.0 (GraphPad Software, La Jolla, CA, USA).

## Results

### Baseline characteristics

No significant differences existed in age, cyst size, gravidity, parity, infertility, dysmenorrhea, r-AFS Staging, blood loss volume, and operation time between the two groups. Serum progesterone was significantly higher in the late luteal phase than in the early follicular phase on the day of surgery ([2.46 ± 1.43] vs. [0.43 ± 0.34]; *P* < 0.001, Table 1). After laparoscopy, no severe deep-infiltrating endometriosis was observed in this study. Postoperative pathological diagnosis proved that all patients had ovarian endometrioma, consistent with the preoperative diagnosis.

**Table 1:** Clinical characteristics of the study subjects

Variable	LLP (n = 44)	EFP (n = 44)	*P-*value
Age, (years)	30.71 ± 3.46	29.36 ± 3.15	0.058
Age of menarche, (years)	13.32 ± 1.16	13.02 ± 1.19	0.245
Endometrioma volume, (mm^3^)	117.71 ± 13.97	117.93 ± 15.79	0.954
Pre-operation serum E2, (pg/mL)	89 ± 68.26	90 ± 47.78	0.834
Pre-operation serum P, (ng/mL)	2.46 ± 1.43	0.43 ± 0.34	< 0.001***
Gravidity, n (%)			
0	24(54.54%)	24 (54.54%)	1
≥ 1	20(45.45%)	20 (45.45%)	
Parity, n (%)			
0	24(54.54%)	26 (59.09%)	0.674
≥ 1	20(45.45%)	18 (40.90%)	
Infertility, n (%)	15	16	0.823
Dysmenorrhea, n (%)	13(29.54%)	16 (36.36%)	0.501
r-AFS Staging, n (%)			
I-II	0	0	/
III	18(40.90%)	21 (47.73%)	0.524
IV	26(59.09%)	23 (52.27%)	
Blood loss, (mL)	43.86 ± 9.72	43.86 ± 21.83	1
Operation time, (min)	65.82 ± 9.21	67.73 ± 10.26	0.355

***: *P* < 0.01. **LLP**: Late luteal phase; **EFP**: Early follicular phase; **E2**: Estradiol; **P**: Progesterone

Data are presented as mean ± SD or n (%). a: Groups compared by t-test; b: Groups compared by Pearson’s chi-squared test or Fisher’s exact test;

### AMH as the biomarker to evaluate an ovarian reserve and follicle loss

There was no significant difference in preoperative AMH between the two groups. The serum AMH values one week after surgery were higher in group LLP than that in group EFP ([3.58 ± 1.65] vs. [3.02 ± 1.22], *P =* 0.075). However, AMH decrease value was significantly lower than that of group EFP ([1.31 ± 0.93] vs. [1.87 ± 0.97], *P* = 0.007). Serum AMH at postoperative six months in group LLP was significantly higher than that in group EFP ([3.35 ± 1.67] vs. [2.61 ± 1.15], *P* = 0.018). In contrast, AMH decrease values at postoperative six months were significantly higher in group EFP than that in group LLP ([1.54 ± 0.93] vs. [1.91 ± 1.06]; *P* < 0.001, Table 2). The mean rates of postoperative serum AMH decline were 37.92% and 46.34% in group EFP, respectively, significantly higher than those of group LLP (25.83% vs. 31.43%) (*P*<0.001, Table 3).

**Table 2:** Serum AMH levels of pre-operation and post-operation in two groups

Variable	Pre-operation AMH (ng/mL)	1 Week-Po AMH (ng/mL)	DV1	6 months-Po AMH (ng/mL)	DV6
LLP (n = 44)	4.89 ± 2.37	3.58 ± 1.65	1.31 ± 0.93	3.35 ± 1.67	1.54 ± 0.93
EFP (n = 44)	4.89 ± 1.56	3.02 ± 1.22	1.87 ± 0.97	2.61 ± 1.15	1.91 ± 1.06
*P-*value	1	0.075	0.007**	0.018**	0.001**

**LLP**: Late luteal phase; **EFP**: Early follicular phase; Po: post-operation; **AMH**: anti-Müllerian hormone; **DV**: Difference Value, **DV1**: Difference between preoperative AMH and one-week postoperative AMH; **DV6**: Difference between preoperative AMH and six months postoperative AMH.

**: *P* < 0.05

**Table 3:** Decrease rate of serum AMH levels of post-operation

Variable	Decrease rate of AMH 1 Week-Po (%)	Decrease rate of AMH 6 months-Po (%)
LLP (n = 44)	25.83 ± 10.12	31.43 ± 11.13
EFP (n = 44)	37.92 ± 14.13	46.34 ± 14.23
*P-*value	< 0.001***	< 0.001***

**LLP**: Late luteal phase; **EFP**: Early follicular phase; **AMH**: anti-Müllerian hormone

Decrease rate of AMH 1 Week-Po: Decrease rate of AMH at one-week post-operation compared to preoperative AMH; Decrease rate of AMH six months-Po: Decrease rate of AMH at six months post-operation compared to preoperative AMH; ***: P < 0.01

**Fig. 1:**
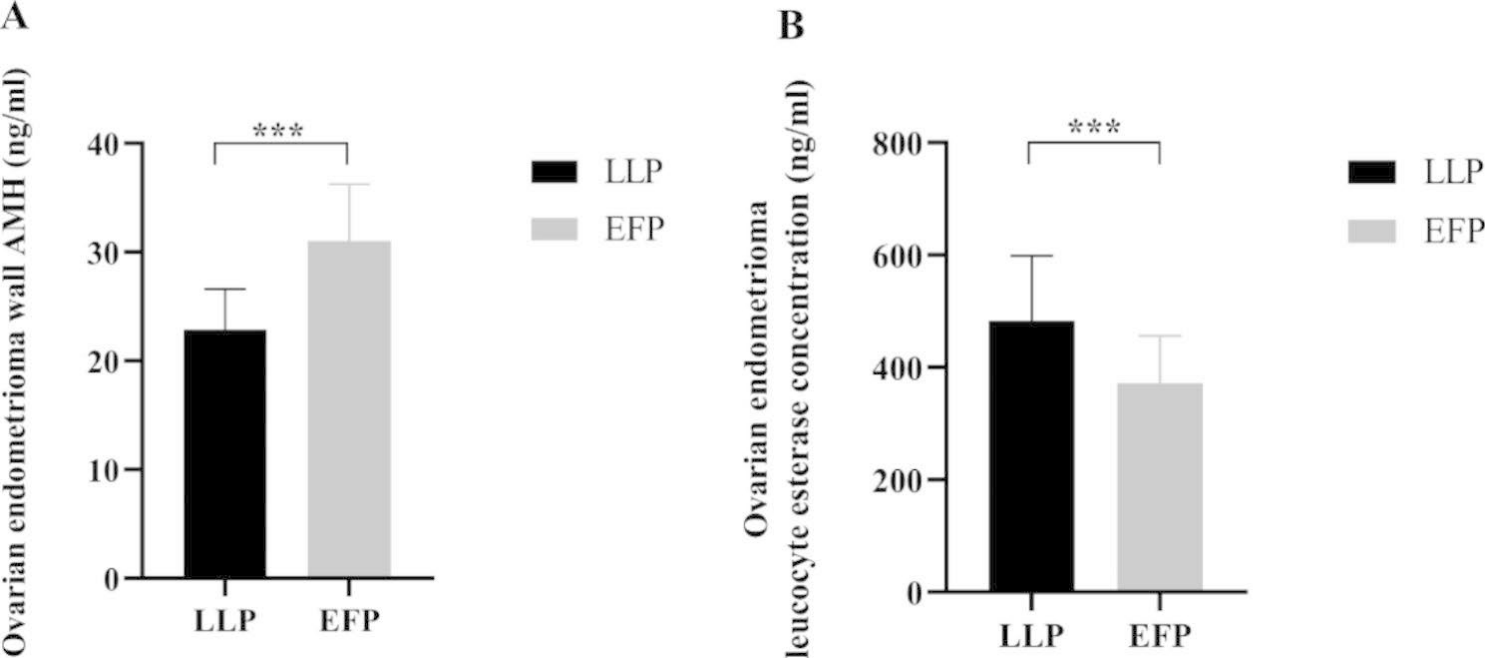
Ovarian endometrioma wall AMH and leucocyte esterase concentration. **LLP**: Late luteal phase; **EFP**: Early follicular phase; **AMH**: anti-Müllerian hormone; ***: *P* < 0.01

**Fig. 2:**
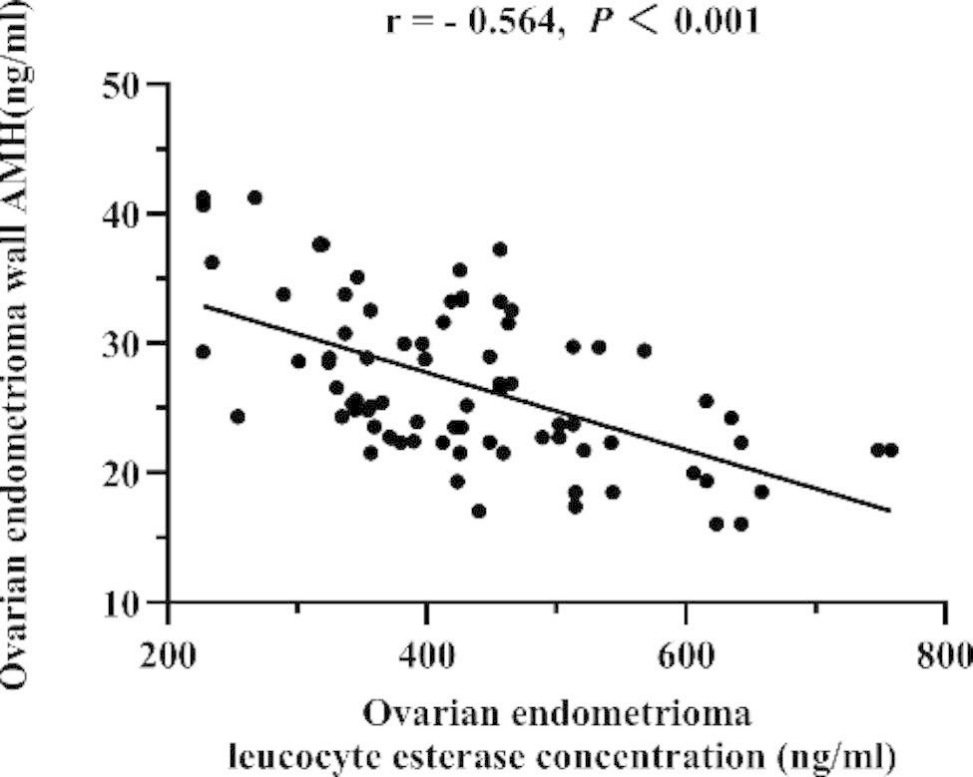
Correlation analysis between ovarian endometrioma wall AMH and leucocyte esterase concentration. AMH: anti-M?llerian hormone; *P* < 0.05 means the difference was statistically significant

Ovarian endometrioma wall AMH of group LLP was significantly lower than that of group EFP ([22.86 ± 3.74] vs. [31.02 ± 5.23], *P*<0.001). Meanwhile, ovarian endometrioma leucocyte esterase concentration of group LLP was significantly higher than that of group EFP ([482.83 ± 115.88] vs. [371.68 ± 84.49], *P*<0.001, Table 4; Fig. 1). Moreover, significant negative correlation exists between LE and AMH concentration in the cyst wall of ovarian endometrioma (*P*<0.001, Fig. 2).

**Table 4:** Ovarian endometrioma wall AMH and leucocyte esterase concentration

Variable	Ovarian endometrioma wall AMH (ng/mL)	Ovarian endometrioma leucocyte esterase concentration (ng/mL)
LLP (n = 44)	22.86 ± 3.74	482.83 ± 115.88
EFP (n = 44)	31.02 ± 5.23	371.68 ± 84.49
*P-*value	< 0.001***	< 0.001***

**LLP**: Late luteal phase; **EFP**: Early follicular phase; **AMH**: anti-Müllerian hormone; ***: *P* < 0.01

## Discussion

Laparoscopic endometrioma cystectomy is a recommended and widely used method because it meets the diagnostic and treatment goals of endometriosis, which can reduce pain, increase the chance of spontaneous pregnancy, and reduce disease progression and recurrence [19–21]. However, in addition to the possible negative effect of endometriosis on ovarian reserve, serum AMH levels significantly decrease after laparoscopic cystectomy for endometrioma [5, 22–26]. Since the ovarian reserve responds to the woman’s reproductive function, it must be preserved ultimately during laparoscopic cystectomy.

Anti-Müllerian Hormone (AMH) is a transforming growth factor-β family member secreted by primary, preantral, and antral follicles [27]. AMH levels correlate with the number of growing follicles and do not change significantly during the menstrual cycle [14, 28]. Therefore, AMH has been used to predict the decline of ovarian function and is the preferred biomarker of ovarian reserve [29, 30]. Several hypotheses have been formulated to explain the relationship between cyst excision and reduction of ovarian reserve. Some authors demonstrated that the removal of ovarian endometrioma, commonly characterized by the absence of a clear plane of cleavage between the endometrioma cyst and ovarian tissue, could result in unintentional removal of the ovarian cortex and loss of follicles, with a potential reduction in follicular reserve [31, 32]. Furthermore, the amount of ovarian parenchyma loss seems to increase proportionally to the increase in cyst diameter [33]. According to this hypothesis, damage to the ovarian reserve can result from permanent loss of ovarian tissue and should persist over time after surgery [23]. This study investigated the optimal surgical timing to perform a cystectomy. It can reduce ovarian function damage by evaluating serial changes in serum AMH levels after laparoscopic endometriosis cystectomy for endometriosis and evaluating ovarian endometrioma wall AMH and ovarian endometrioma leucocyte esterase concentration.

Our results displayed that serum AMH levels decreased significantly at one week and six months after surgery. However, the decreasing trend of serum AMH levels in group LLP was significantly lower than in group EFP. A systematic review and meta-analysis showed that the median preoperative AMH level was 3.1 ng/mL, which significantly decreased to 1.51 ng/mL within 1–9 months after surgery, with a decline rate of 51.29% [3]. A prospective longitudinal study showed that the rate of decrease in AMH was 52.2%, 53.7%, and 54.8% at 1, 3, and 6 months after surgery compared to baseline levels, respectively [34]. In this study, compared with baseline levels, patients who underwent surgical treatment in the late luteal phase had AMH decline rates of 25.8% and 31.4% at one week and six months postoperatively, respectively. However, patients treated surgically at the early follicular phase had AMH decline rates of 37.9% and 46.3% at one week and six months postoperatively, respectively. Hoang Tong et al. found that unilateral ovarian cystectomy with a 43.4–48% decrease in serum AMH from 1 to 6 months after surgery was the same as the cystectomy results performed in the early follicular phase in this study [34]. Zhou Liu et al. estimated the distance to restore ovarian reserve after laparoscopic unilateral ovarian cystectomy to be six months [35]. Urman et al. found a significant decrease in AMH concentration and antral follicle count (AFC) one month after surgery, a reduction that persisted six months postoperatively [36]. Therefore, we conclude that laparoscopic cystectomy for unilateral ovarian endometrioma at the late luteal phase may reach a content result about follicle loss and ovarian reserve.

AMH is produced by granulosa cells of primary, preantral, and small antral follicles. Although there have been few studies on detecting ovarian endometrioma wall AMH, we believe it is closely related to the number of granulosa cells in the endometrioma wall, reflecting the quantity of ovarian tissue and follicles in the endometrioma wall. Leukocyte esterase activity is commonly used for sensitive detection of leukocytes. Leukocytes infiltrate the ovarian endometrioma wall, resulting in endometrioma wall edema and loose tissue. Our study also found that ovarian endometrioma wall AMH of group LLP in patients with first ovarian cystectomy was significantly lower than that of group EFP ([22.86 ± 3.74] vs. [31.02 ± 5.23], *P*<0.001). In contrast, the ovarian endometrioma leucocyte esterase concentration of group LLP was significantly higher than that of group EFP ([482.83 ± 115.88] vs. [371.68 ± 84.49], *P*<0.001). A significant inverse relationship was observed between leukocyte esterase and AMH concentration in the ovarian endometrioma cyst wall (*P* < 0.001). Our results revealed that ovarian cystectomy with different menstrual cycles may affect the rate of decrease in AMH levels after laparoscopic ovarian cystectomy in patients with endometriosis. The border density between endometrioma and normal ovarian tissue may fluctuate during the menstrual cycle. Loosening of the border and inflammatory edema of the tissue allows the cyst wall to be more easily peeled off in the late luteal phase, reducing the loss of normal ovarian tissue.

This study had some limitations. First, this study analyzed data from one single center. Second, this study was conducted only in patients with the first identified single ovarian endometrioma. The results were unavailable for patients with previous endometrioma and pelvic surgery. Third, patients with bilateral ovarian endometrioma cysts were not investigated. Finally, this study had a short follow-up period (six months). We used oral contraceptive to control the menstrual cycle to ensure that surgery was performed in the late luteal phase. The specific mechanism needs to be further studied. Whether surgery can achieve the same effect after the withdrawal of other exogenous progesterone is worth studying.

In conclusion, our findings suggest that laparoscopic cystectomy in the late luteal phase is an advantageous option for patients with endometrioma, as it has been shown for the first time to effectively and safely reduce ovarian tissue loss and preserve ovarian reserve. We also recommend that once the endometriosis cyst is diagnosed and laparoscopic surgery is proposed, OC should be performed to control the menstrual cycle immediately and inhibit disease progression. In addition, drug therapy should be continued after surgery to achieve efficient disease management and protect ovarian function as far as possible. However, more prospective studies, longer follow-ups, and multiple-center data are required to support clinical practice and underlying mechanisms.

## Data Availability

Data from this study are publicly unavailable owing to ethical and legal restrictions. However, data may be made available upon reasonable request to the corresponding author.

## Declarations

### Provenance and peer review

Not commissioned, externally peer-reviewed.
